# The beneficial effects of *Lacticaseibacillus casei* on the small intestine and colon of Swiss mice against the deleterious effects of 5-fluorouracil

**DOI:** 10.3389/fimmu.2022.954885

**Published:** 2022-10-21

**Authors:** Stphannie Jamyla de Araújo Barbosa, Maisie Mitchele Barbosa Oliveira, Susana Barbosa Ribeiro, Caroline Addison Carvalho Xavier de Medeiros, Maria Laura de Souza Lima, Gerlane Coelho Bernardo Guerra, Raimundo Fernandes de Araújo Júnior, Francisco Caninde de Sousa Junior, Agnes Andrade Martins, Daniel Felipe Fernandes Paiva, Raphael Victor Silva Andrade, Conceição S. Martins Rebouças, Gerly Anne de Castro Brito, Renata Ferreira de Carvalho Leitâo, Aurigena Antunes de Araújo

**Affiliations:** ^1^ Postgraduate Program in Pharmaceutical Science, Department of Biophysical and Pharmacology, Federal University of Rio Grande Norte, Natal, Brazil; ^2^ Postgraduate Program in Biotechnology /RENORBIO, Department of Biophysical and Pharmacology, Federal University of Rio Grande Norte, Natal, Brazil; ^3^ Department of Biophysical and Pharmacology, Federal University of Rio Grande Norte, Natal, Brazil; ^4^ Postgraduate Program in Biochemistry and Molecular Biology, Department of Biophysical and Pharmacology, Federal University of Rio Grande Norte, Natal, Brazil; ^5^ Postgraduate Program in Oral Sciences, Department of Biophysical and Pharmacology, Federal University of Rio Grande Norte, Natal, Brazil; ^6^ Postgraduate Program in Health Sciences, Department of Morphology, Federal University of Rio Grande Norte, Natal, Brazil; ^7^ Postgraduate Program in Functional and Structural Biology UFRN, Department of Morphology, Federal University of Rio Grande Norte, Natal, Brazil; ^8^ Postgraduate Program in Pharmaceutical Science, Department of Pharmaceutical Science, Federal University of Rio Grande Norte, Natal, Brazil; ^9^ Department of Dentistry, Federal University of Rio Grande Norte, Natal, Brazil; ^10^ Department of Morphology, Federal University of Ceará, Fortaleza, Brazil

**Keywords:** chemotherapy, 5-fluorouracil, mucositis, probiotic, *Lacticaseibacillus casei*

## Abstract

**Background:**

Intestinal mucositis is one of the most common and important side effects of 5-fluorouracil (5-FU). Currently, there are still no specific and effective protocols for its prevention and treatment. The aim of the present study was to evaluate the effect of oral administration of Lacticaseibacillus casei (*L. casei*) on the progression of 5-FU-induced intestinal mucositis. Methods: *L. casei* (1x10^9^ CFU/ml) or saline was orally administered to Swiss mice, beginning 15 days before intestinal mucositis induction by single intraperitoneal 5-FU administration (450 mg/kg). Body weight, number of peripheral leukocytes and fecal lactic acid bacteria were monitored. After euthanasia, on day 18, tissue samples from colon and each small intestine segment were collected for histopathology. Jejunal tissues were collected and evaluated for iNOS and TNF-alpha immunoexpression, IL-1-beta, IL-6 and TNF-alpha levels, malonaldehyde (MDA) accumulation, invertase activity and factor nuclear kappa B (NFkB-P65) gene expression, toll like receptor-4 (TLR-4), mucin-2 (MUC-2), occludin and zonula occludens-1 (ZO-1).

**Results:**

The positive impact of *L. casei* on 5-FU-induced leukopenia was observed, but not on 5-FU-induced weight loss in mice. *L. casei* reduced 5-FU-induced inflammation in the colon and small intestine (p<0.05). Decreased TNF-α, IL-1β, IL-6 (p<0.05) and MDA (p<0.05) levels, as well as decreased iNOS and TNF-alpha protein expressions (p<0.05) were found in the jejunum from *L casei* group. In addition, *L-casei* down-regulated NFKB-P65 (p<0.05) and TLR-4 (p<0.05) gene expressions and up-regulated MUC-2 and mucosal barrier proteins occludin and ZO-1 gene expressions (p<0.05). Furthermore, greater lactic acid bacteria population (p<0.05) was found in the *L. casei* group when compared to control groups.

**Conclusion:**

Oral *L. casei* administration can protect the intestine of Swiss mice from 5-FU-induced intestinal mucositis, thus contributing to overall health.

## Introduction

5-Fluorouracil (5-FU) is a widely used anticancer drug. It has played an important role in the treatment of colon cancer and is used for patients with breast and other cancers, like those of head and neck. However, approximately 80% of patients undergoing 5-FU treatment suffer from gastrointestinal mucositis (1).

Mucositis can be characterized by three phases (inflammation, epithelial degradation and ulceration), which leads to rapid loss of bowel structure and functionality (2) and presents a series of clinical manifestations such as pain when swallowing, loss of appetite, vomiting, abdominal distention, diarrhea, in addition to changes in the intestinal barrier, increasing its permeability (3).

At present, there are no well-established therapeutic strategies available for the management of intestinal mucositis. Thus, the use of probiotics as an effective intervention against chemotherapy-induced mucositis has been investigated (4).

Probiotics are “live microorganisms that, when administered in adequate amounts, provide health benefits to the host” (5). They are able to restore the intestinal microbiota balance upon the occurrence of inflammation and also stimulate both specific and non-specific immune responses (6). The investigation of probiotics as a therapeutic approach in 5-FU-induced intestinal mucositis is justified by the fact that 5-FU treatment alters the diversity and the community composition of the gut microbiota (7).

In fact, the literature has reported the positive effects of gram-positive lactic acid-producing bacteria of the genus Lactobacillus on experimental chemotherapy-induced mucositis, preventing weight loss, diarrhea and intestinal ulcers (8). It has been shown that the *L. casei* BL23 strain is able to reduce inflammation associated to 5-FU-induced mucositis in mice (9). However, further studies are needed to elucidate the mechanisms by which probiotics exert beneficial effects, which include the preservation of the intestinal barrier, as reported in literature (10).

Therefore, in order to investigate a novel strategy to prevent 5-FU-induced-intestinal mucosite, the effect of oral *Lacticaseibacillus casei* administration on an experimental intestinal mucositis model in mice was investigated.

## Materials and methods

### *Lacticaseibacillus casei* preparation

*Lacticaseibacillus casei* (*L. casei)* probiotic was provided by Farmafórmula LTDA-Farmácia Manipulação, a recognized Brazilian supplier of medicines and supplements, whose products are regulated by the new legal guidelines for food supplements from the National Health Surveillance Agency (ANVISA). The strain was deposited on the China General Microbial Culture Collection Management Center, CGMCC No 1.5409. The viability of freeze-dried *L. casei* was evaluated by plating on MRS agar, according to methodology described by Ribeiro et al. (11). For *L. casei* treatment, freeze-dried *L. casei* at concentration of 2 x 10^10^ CFU/g was suspended using sterile water and diluted to obtain concentration of 1 × 10^9^ CFU/mL.

### Animals and sample size

Female Swiss mice (*Mus musculus*), weighing 25–30 g (mean age of 8 weeks) were housed in polypropylene boxes and kept under controlled temperature (24 ± 2 °C) and relative air humidity conditions (50 ± 5%), 12 h light/dark cycle and *ad libitum* access to food and water. Animals were obtained from the animal facilities of the *Biosciences Center - Federal University of Rio Grande do Norte* (*UFRN)*, Brazil. All experimental protocols were approved by the Ethics Committee on the use of Animals (CEUA) of the Federal University of Rio Grande do Norte (Protocol No.017/2019) and performed in accordance with the ARRIVE ethical guidelines. All methods were performed in accordance with relevant guidelines and regulations.

The number of animals per group was determined according to the sample size formula n = DF/k + 1, which can be used for three common ANOVA designs applicable to animal studies, where k = number of groups, n = number of subjects per group, and DF= degrees of freedom (12). In view of the ethical considerations that recommend the sample size refinement in studies with animals, we chose to use the minimum number of animals (5 animals/group) to carry out experiments.

### Experimental intestinal mucositis

Intestinal mucositis was induced as previously described (8). Briefly, a single dose of 450 mg/kg of 5-Fluorouracil (Libbs Pharmaceuticals Ltd. (São Paulo, Brazil)) was intraperitoneally administered and animals were euthanized 3 days later by overdose of ketamin (240mg/kg) and xylazin (30mg/kg).

### Experimental and control groups

To investigate the impact of *L. casei* on 5-FU-induced intestinal mucositis, a group of 5 animals received once-daily oral administration of *L. casei* (1x10^9^ CFU/ml), starting 15 days before the 5- FU administration, until euthanasia, 3 days after the 5-FU injection, on day 18 (*L. casei* group). The control animals were divided into two control subgroups (n=5/group): a group of healthy animals, not submitted to 5-FU-induced intestinal mucositis, that received once-daily administration of saline solution until euthanasia and a single intraperitoneal injection of saline solution on day 15 (saline group) and a group of animals submitted to 5-FU-induced intestinal mucositis that received once-daily administration of saline solution from day 0 to day 18 (5-FU group). All animals were euthanized 4 days after the 5-FU injection, on day 19.

The animals were monitored daily up to 18th day for signs of moribundity and mortality, such as lack of responsiveness to manual stimulation; immobility; and/or an inability to eat or drink.

### Body weight and peripheral blood leucocyte counts

In an attempt to detect possible systemic 5-FU-associated toxicity, body weight (measured on days 1 and 18) and white blood cell counts were investigated. Immediately before euthanasia, blood samples (20 μL), collected from heart puncture of anesthetized animals, was diluted in 380 μL of Turk’s solution. The total leukocytes were counted manually using a Neubauer chamber and the results are expressed as the number of white blood cells per mm^3^ of blood.

### Histopathological analysis

Following euthanasia, tissue samples (including the mucosal, submucosal, muscle, and serosa layer) of each small intestine sections (duodenum, jejunum and ileum) and colon were collected and fixed in 10% neutral-buffered formalin, dehydrated, and embedded in paraffin for immunohistochemistry and histopathological analysis. Sections (5 µm thick) were obtained for hematoxylin and eosin staining (H&E) and for subsequent light microscopy examination (200x). The severity of mucositis was evaluated in a single-blinded fashion and was graded using a modification of the Macpherson and Pfeiffer histopathological grading system (13). *Score 0*: Normal histological findings; *Score 1*: Mucosa: villus blunting, loss of crypt architecture, sparse inflammatory cell infiltration, vacuolization and edema. Normal muscular layer; *Score 2*: Mucosa: villus blunting with fattened and vacuolated cells, crypt necrosis, intense inflammatory cell infiltration, vacuolization and edema. Normal muscular layer; *Score 3:* Mucosa: villus blunting with fattened and vacuolated cells, crypt necrosis, intense inflammatory cell infiltration, vacuolization and edema. Muscular: edema, vacuolization, sparse neutrophil infiltration.

### Invertase activity

For the assay of invertase activity in the jejunal tissues, the 3,5-dinitrosalicylic acid (DNS) method was used, as previously described by Araujo et al. (14). To generate a standard curve, a stock solution of 1 g/L glucose was prepared, and 1 mL, 0.8 mL, 0.6 mL, 0.4 mL, and 0.2 mL aliquots of this solution were added to five tubes, followed by the addition of 0 mL, 0,2 mL, 0.4 mL, 0.6 mL, and 0.8 mL distilled water to achieve the following concentrations of glucose: 1 g/L, 0.8 g/L, 0.6 g/L, 0.4 g/L, and 0.2 g/L. In addition, a sixth tube contained 1 mL distilled water. DNS (3,5-dinitro-2-hydroxybenzoic acid, Sigma-Aldrish, São Paulo, Brazil) reagent (0.5 mL) was added to each test tube. The tubes were heated in a 100°C water bath for 5 min, and then were transferred to a cold-water bath. Distilled water (8.5 mL) was added to each tube (total volume, 10 mL), and the percent transmittance values at 540 nm were recorded. The results are *expressed* as the *amount* of *enzyme* required to *release* one µmol of reducing *sugar* in one *minute*.

### Malondialdehyde dosage

The content of MDA, a product of lipid peroxidation, was investigated in the jejunal tissue samples (n=5/group) as a marker of oxidative stress, as previously described (15). Briefly, samples were suspended in Trizma 1:5 (w/v) buffer. The material was incubated for 40 minutes at 45°C in a water bath, centrifuged at 2500 G for 5 minutes at 4°C; 300 μL was then removed, read at 586 nm, and interpolated in a standard curve. Supernatants were tested for MDA content and placed in microplates. The absorbance of each sample was measured at 586 nm. The results are expressed as nanomoles of MDA per ml of plasma or mg of tissue.

### Immunohistochemical analysis

Sections (4 µm thick) were prepared from parafin-embedded intestinal tissues. After deparaffinization, antigens were recovered by incubating the slides in citrate buffer (pH 6.0) for 20min at 95°C. Endogenous peroxidase was blocked with 3% H_2_O_2_ for 10 min to reduce nonspecific binding. Sections were then incubated with TNF-α (ab270264, Abcan) or iNOS (ab3523, Abcan) for 2h. Sections were then incubated for 30 min with polymer (K4061, DAKO). Antibody binding sites were visualized by incubating the samples with diaminobenzidine–H2O2 (DAB, DAKO) solution. Sections incubated with antibody diluent, without a primary antibody were used as negative controls. The amounts of DAB products from immunostaining were estimated from digital images of at least ten different areas of each section (from 4 specimens per group) at 400x magnification using Adobe Photoshop sofware. The results are expressed as the percentage of immunopositive area, calculated by dividing the DAB-positive staining (immunostaining-positive pixels) by the number of pixels per total tissue image multiplied by 100, as previously described (16).

### Cytokine assay (IL-1β, IL-6, TNF-α)

The IL-1β, IL-6 and TNF-α levels were determined from jejunum samples (n=5/group), which were stored at − 80 °C until required for this assay. The samples were homogenized and processed as previously described (17). The concentrations of IL-1β, IL-6 and TNF-α in the samples were determined using a commercial ELISA kit (R&D Systems, Minneapolis, MN, USA). Briefly, microtiter plates were coated overnight at 4 °C with antibodies against IL-1β (detection range: 62.5–4000 pg/mL; sensibility or lower limit of detection: 12.5 ng/mL of recombinant mouse IL-1β), IL-6 (detection range:125–8000 pg/mL) and TNF-α (detection range: 62.5–4000 pg/mL; sensibility or lower limit of detection: 50 ng/mL of recombinant mouse TNF-α). After blocking the plates, the samples and standard at various dilutions were added in duplicate and incubate at 4 °C for 24 h. After washing the plates (three times with buffer) biotinylated polyclonal anti-IL-1β or anti-TNF-α, diluted 1:1000 with assay buffer 1% BSA, was added to the wells. After further incubation at room temperature for 1 h, the plates were washed and streptavidin-HRP, diluted 1:5000, was added to each well. The chromogenic reagent O-phenylenediamine was added 15 min later and the plates were incubated in the dark for 15 min. The enzymatic reaction was interrupted with H_2_SO_4_ and the absorbance was measured at 490 nm using UV–VIS spectrophotometry. The results are expressed as pg/mL (18).

### RT-PCR gene marker analysis

The ribonucleic acid (RNA) was isolated from jejunum segments (n=5/group), using Trizol reagent (Invitrogen, Carlsbad, CA, USA) according to the manufacturer’s instructions. RNA was quantified by NanoDrop and the samples purity was verified by 260/280 ratios >1.8. Five micrograms of isolated total RNA (10ul) was transcribed to cDNA in a reaction mixture containing 2 ul 10X RT buffer, 0.8 ul 25X dNTP Mix, 2ul 10X RT oligo dT, 1ul MultiScribe Reverse transcriptase 4,2ul H20 (High-Capacity cDNA Reverse Transcription Kit, Foster City, CA, USA) in a total volume of 20 μL. The reaction mixture was incubated at 25°C for 10 min, 37ªC for 120 min, 85°C for 5min and 4°C for ∞.

The cDNA was stored at −80°C until further use. qPCR was performed using SYBR Green PCR Master Mix (Applied Biosystems), as described in the manufacturer’s instructions. The sequences of the primers are listed in **Table 1**. To compare gene expression under different conditions, the expression under each condition (normalized to ACTB, the endogenous control) was quantified relative to the control condition. qPCR amplification was performed in a CFX Connect system (Bio-Rad) under the following conditions: 50°C for 2 min and 95°C for 10 min, followed by 40 cycles of 95°C for 15 s and 60°C for 60 s. The relative expression levels of the genes were calculated using the threshold cycle (2^−ΔΔCT^) method (19).

**Table 1:** Gene sequence forward and reverse (ACTB, NFKB-P65, TLR-4, MUC-2, OCLN, ZOI).

Primer	Sequence	
ACTB	Foward	AGGCCAACCTGTAAAAGATG
	Reverse	TGTGGTACGAGAGGCATAC
NFkB P65	Foward	CCGTCTGTCTGCTCTCTCT
	Reverse	CGTAGGGATCATCGTCTGCC
TLR-4	Foward	GCCTTTCAGGGAATTAAGCTCC
	Reverse	AGATCAACCGATGGACGTGTAA
MUC-2	Foward	GATAGGTGGCAGACAGGAGA
	Reverse	GCTGACGAGTGGTTGGTGATTG
OCLN	Foward	AGGGACCCTGACCACTATGA
	Reverse	TCAGCAGCAGCCATGTACTC
ZO-1	Foward	TGGAGATGAGGCTTCTGCTT
	Reverse	GGGGCCTACACTGATCAAGA

### Fecal lactic acid bacteria analysis

The population of fecal lactic acid bacteria (LAB) was determined on the 7th and 18th days after the first probiotic administration. Stool samples were collected immediately after defecation on a clean surface. Samples were dispersed in phosphate buffered saline (PBS; pH 7.0) and homogenized using a glass rod for 5 minute (20). Serial decimal dilutions of samples were made in sterile PBS and plated in triplicate on Petri dishes containing MRS agar. The dishes were incubated at 37°C under anaerobic conditions for 72 hours. The results are expressed as a logarithm of Colony Forming Units per gram of a sample (log_10_ CFU/g), as previously described (11).

### Statistical analysis

Data were analyzed using descriptive (mean and standard deviation) and analytical statistics using parametric tests such as ANOVA, followed by a Bonferroni post-test and non-parametric Kruskal–Wallis test at a 5% significance level (Graph Pad Prism 6.01 software).

## Results

### *L. casei* protects against 5-FU-induced leukopenia, but not against 5-FU-induced weight loss

**Figure 1:**
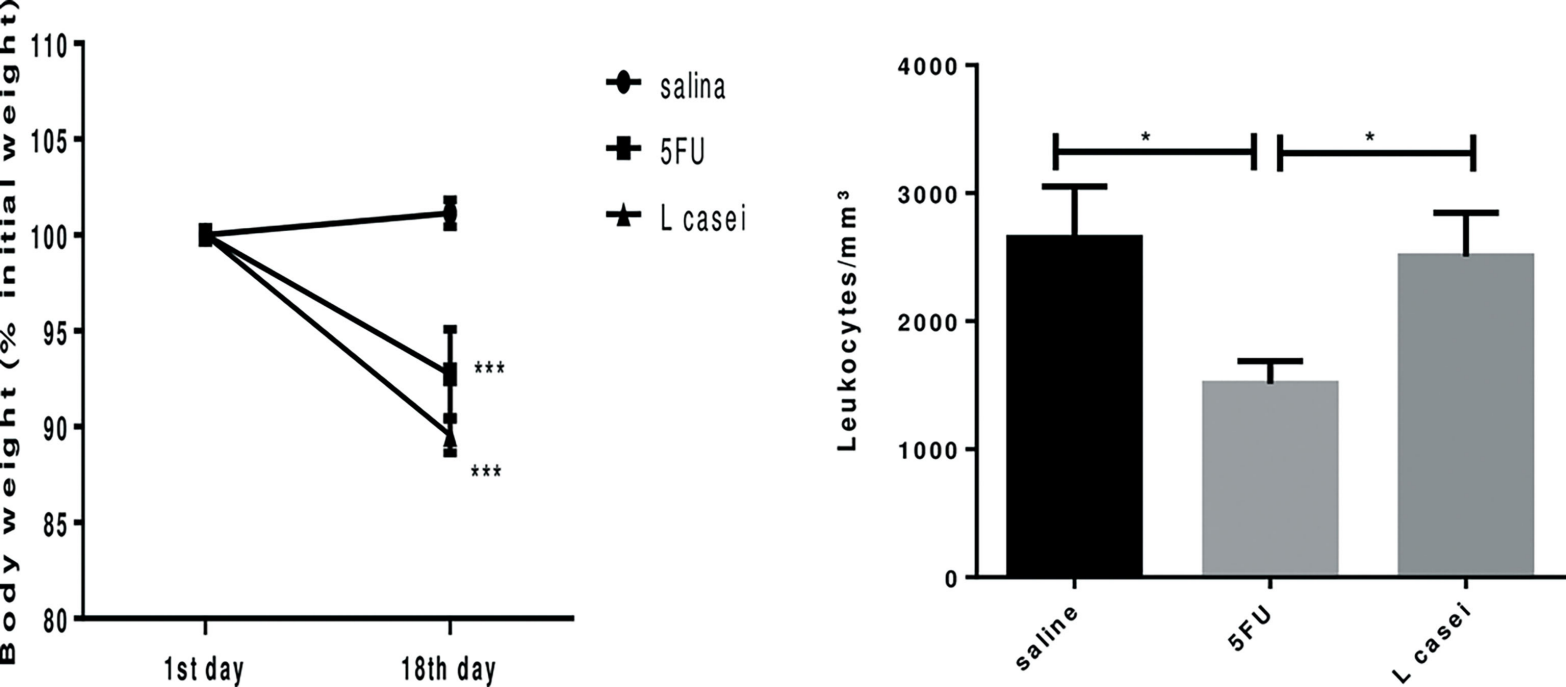
Weight of the animals (1^st^ day and 18^th^ day of treatment) and the leukocyte count (mm³) in the blood after euthanasia. Groups: Saline, 5FU (5-fluorouracil) and *L. Casei* (*Lacticaseibacillus casei*). *p < 0.05, ***p < 0.001.

No animal exhibited signs of cachexia and no mortality was recorded. The prophylactic use of *L. casei* did not have an impact on 5-FU-induced weight loss, as illustrated in **Figure 1**. In addition, **Figure 1** also shows that *L. casei* was able to protect mice against leukopenia (reduction in the number of leukocytes/mm^3^), a cytotoxic effect of 5-FU (p<0.05).

### *L. casei* protects against 5-FU-induced histopathological injury

**Figure 2:**
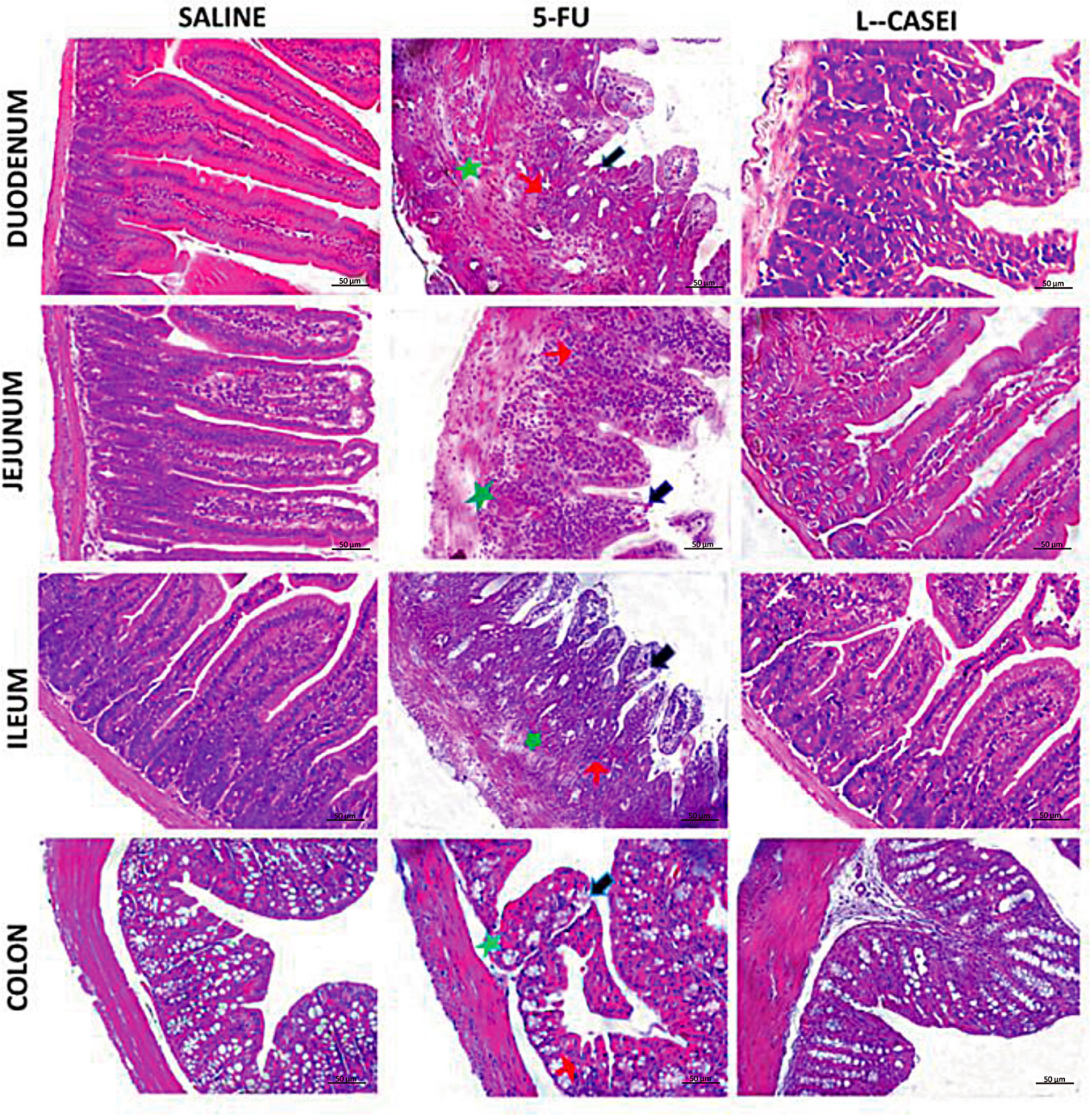
Histophatological analysis. *Lacticaseibacillus* casei (LC), prevented 5-FU-induced histopathological injuries in duodenum, jejunum, ileum and colon. 5-FU induces villi shortening (black arrows), loss of crypt architecture (green arrows) and intense inflammatory cell infiltrate (red arrows) in the duodenum, jejunum, ileum and colon, and edema (brown arrows) in the colon submucosal layer. H&E; scale bar corresponds to 200 µm.

*L. casei* protected the colon and all small intestine segments against 5-FU-induced injury (p<0.05), as shown in **Table 2** and **Figure 2**. 5-FU-induced damage resulted in shortened villi, loss of crypt architecture, submucosal edema, and pronounced inflammatory cell infiltrate in the lamina propria (**Figures 2B, E, H, K**) compared to the saline group (**Figures 2A, D, G, J**). On the other hand, *L. casei* pretreatment was able to attenuate these histological changes promoted by 5-FU in all intestine segments, as shown in **Figure 2**, with preserved villi and crypts in the duodenum (2C), jejunum (2F) and ileum (2I), associated with significantly less edema and less inflammatory cell infiltrate (2C, 2F, 2I and 2L) when compared to the respective 5-FU intestinal segments.

**Table 2:** Histological scores for intestinal mucositis.

Intestinal Segments			
	Control	5-FU	LC
Duodenum	0 (0-0)	2 (2-3)^#^	0 (0-1)^*^
Jejunum	0 (0-0)	3 (3-3)^#^	1 (1-2)^*^
Ileum	0 (0-0)	2 (2-3)^#^	1 (0-1)^*^
Colon	0 (0-0)	2 (2-3)^#^	1 (0-1)^*^

Data represent median values (and range) of scores from 0 to 3: Score 0, normal histological findings; Score 1, villus blunting, loss of crypt architecture, sparse inflammatory cell infiltration, vacuolization and edema normal muscle layer; Score 2, villus blunting with fattened and vacuolated cells, crypt necrosis, intense inflammatory cell infiltration, vacuolization and edema and normal muscle layer; Score 3, villus blunting with fattened and vacuolated cells, crypt necrosis, intense inflammatory cell infiltration, vacuolization and edema and muscle layer showing edema, vacuolization and neutrophilic infiltration. Villus alterations were not considered for colon scores. Data were analyzed with Kruskal-Wallis and Dunn’s tests (n = 5). ^#^P < 0.01 versus control group, *P < 0.01 versus 5-FU group. Lactobacillus casei (LC).

### *L. casei* protects against 5-FU-induced increase in malonaldehyde (MDA) levels, but not against 5-FU-induced reduction of invertase activity

**Figure 3:**
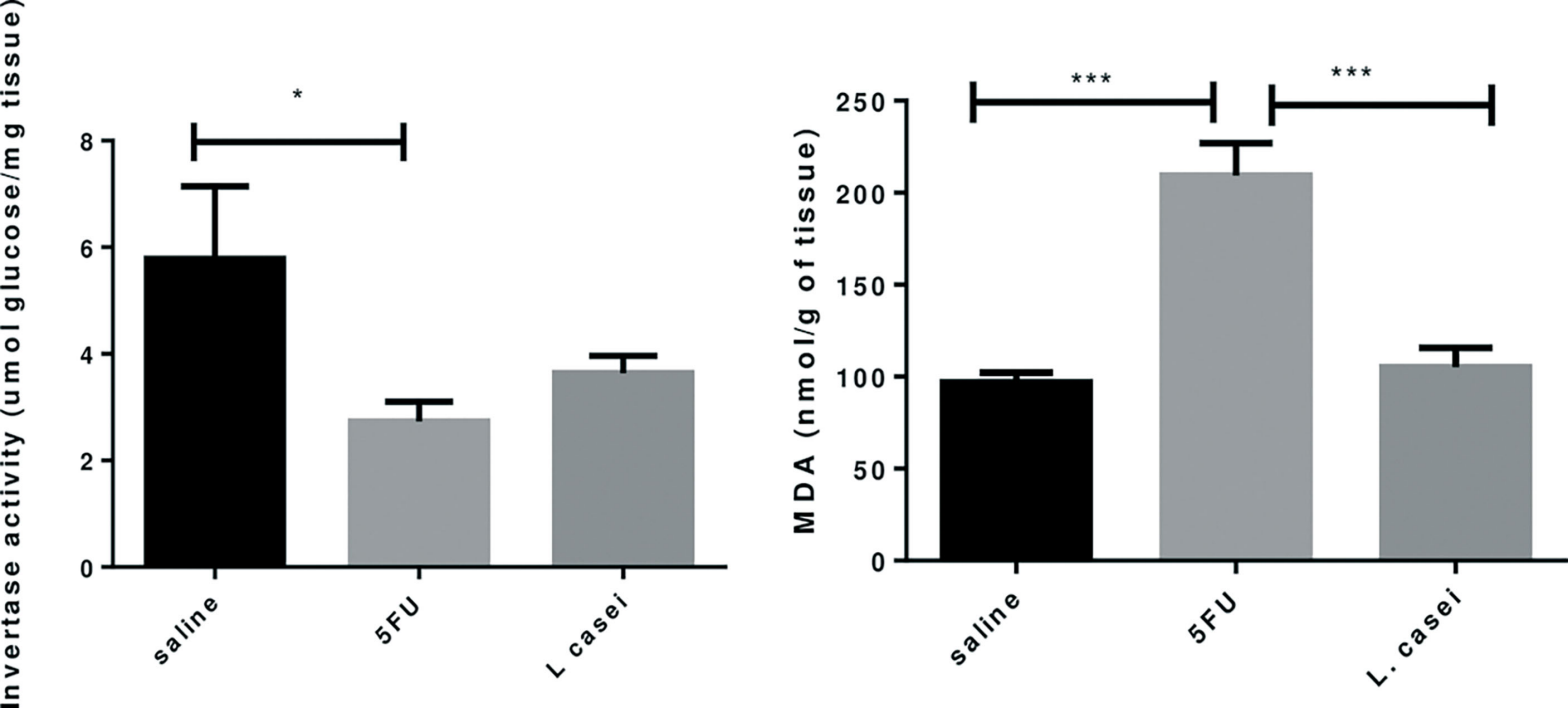
Invertase activity, Malonaldehyde Dosage (MDA). Groups: Saline, 5FU (5-fluorouracil) and *L. Casei* (*Lacticaseibacillus casei*). *p < 0.05, ***p < 0.001.

MDA levels, investigated as a marker of lipid peroxidation oxidative stress in jejunal tissues from the 5-FU group were significant higher when compared to saline group (p < 0.05). *L. casei* was able to protect against this effect of 5-FU, since a significant reduction in MDA levels was found in jejunal tissues of the *L. casei* group when compared to the 5-FU group (p < 0.05), as shown in **Figure 3**.

**Figure 3** also shows that the invertase activity was significantly reduced in the 5-FU group (p<0.05) compared to the saline group. Pretreatment with *L. casei* was not able to preserve the invertase activity, since no significant difference was found between 5-FU and *L. casei* groups (p>0.05), as illustrated in **Figure 3**.

### *L. casei* protects against 5-FU-induced iNOS and TNF-α overexpression

**Figure 4:**
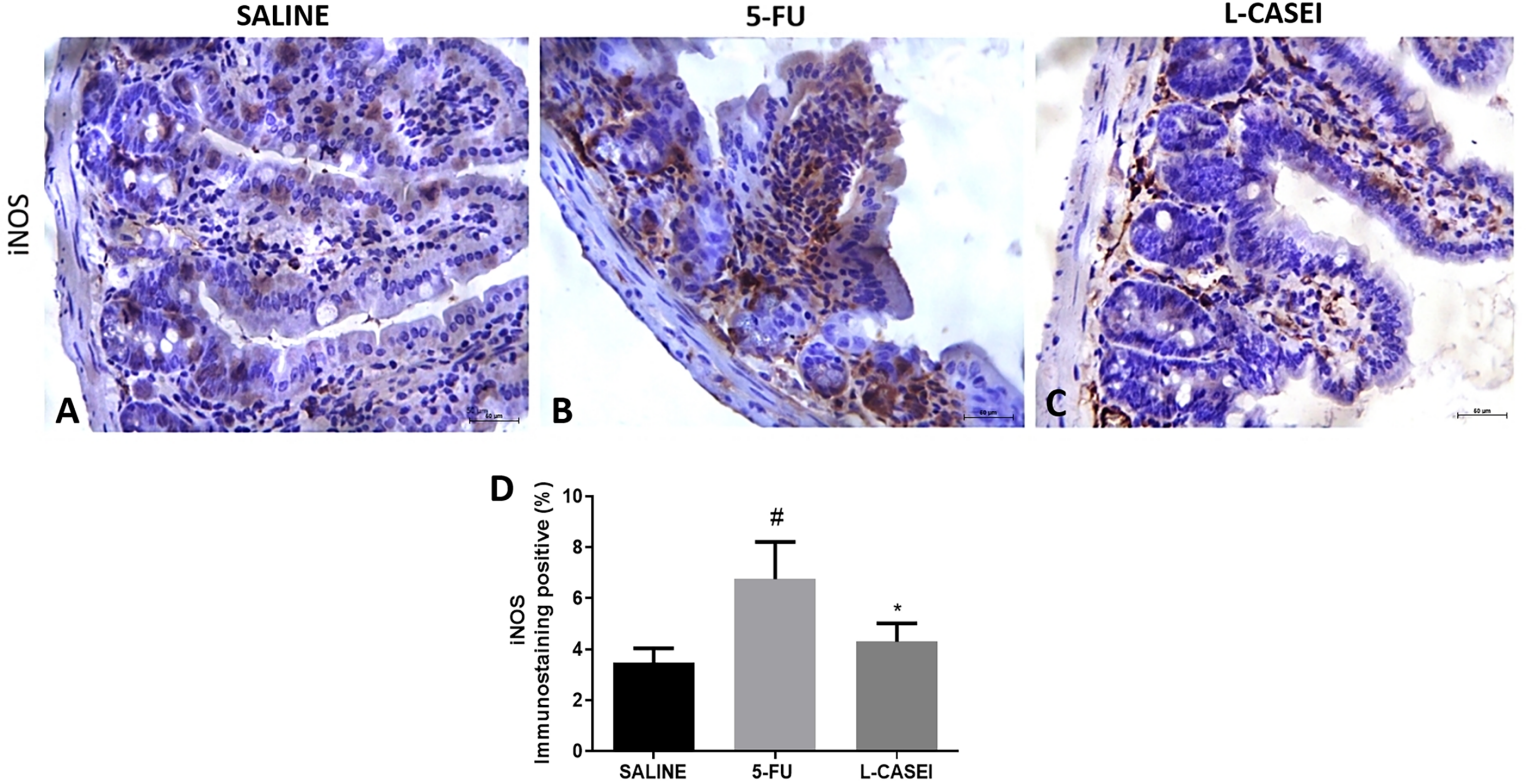
Representing examples of iNOS immunostaining: Control (Saline; **A**), 5-FU **(B)**; L. casei (LC; **C**) and percentage of iNOS immunolabeled cells **(D)**. Values were expressed as mean ± SEM. For statistical analysis, one-way ANOVA was used followed by Tukey's test, where ^#^p < 0.05 vs. control group, *p < 0.05 vs. 5-FU group. Magnification x400.

**Figure 5:**
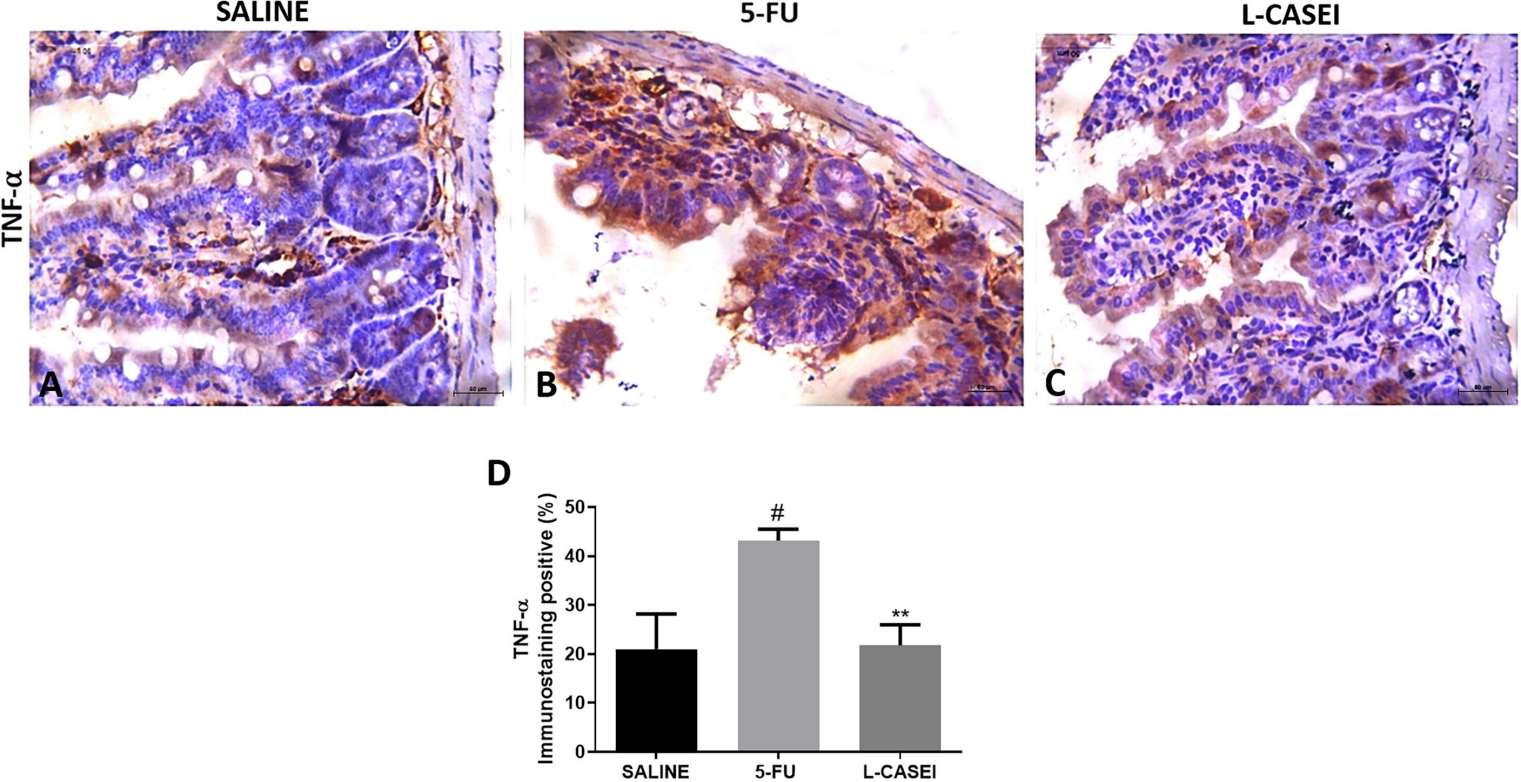
Representing examples of TNF-α immunostaining: Control (Saline; **A**), 5-FU **(B)**; L. casei (LC; **C**) and percentage of TNF-α immunolabeled cells **(D)**. Values were expressed as mean ± SEM. For statistical analysis, one-way ANOVA was used followed by Tukey’s test, where ^#^p < 0.001 vs. control group, **p < 0.01 vs. 5-FU group. Magnification x400.

The jejunal tissues of mice submitted to 5-FU-induced intestinal mucositis showed marked iNOS (**Figures 4B, D**) and TNF-α immunostaining (**Figures 5B, D**) on inflamed conjunctive tissue compared to the saline control group (**Figures 4A, D**, **5A, D**). Pretreatment with *L. casei* significantly reduced (p<0.05) the jejunal iNOS (**Figures 4C, D**) and TNF-α immunostaining (**Figures 5C, D**) when compared to the 5-FU group. No significant differences were found between iNOS and TNF-α immunoexpression between *L.casei* and saline groups (p>0.05).

### *L. casei* protects against 5-FU-induced increase in IL-1 beta, IL-6 and TNF-α levels

**Figure 6:**
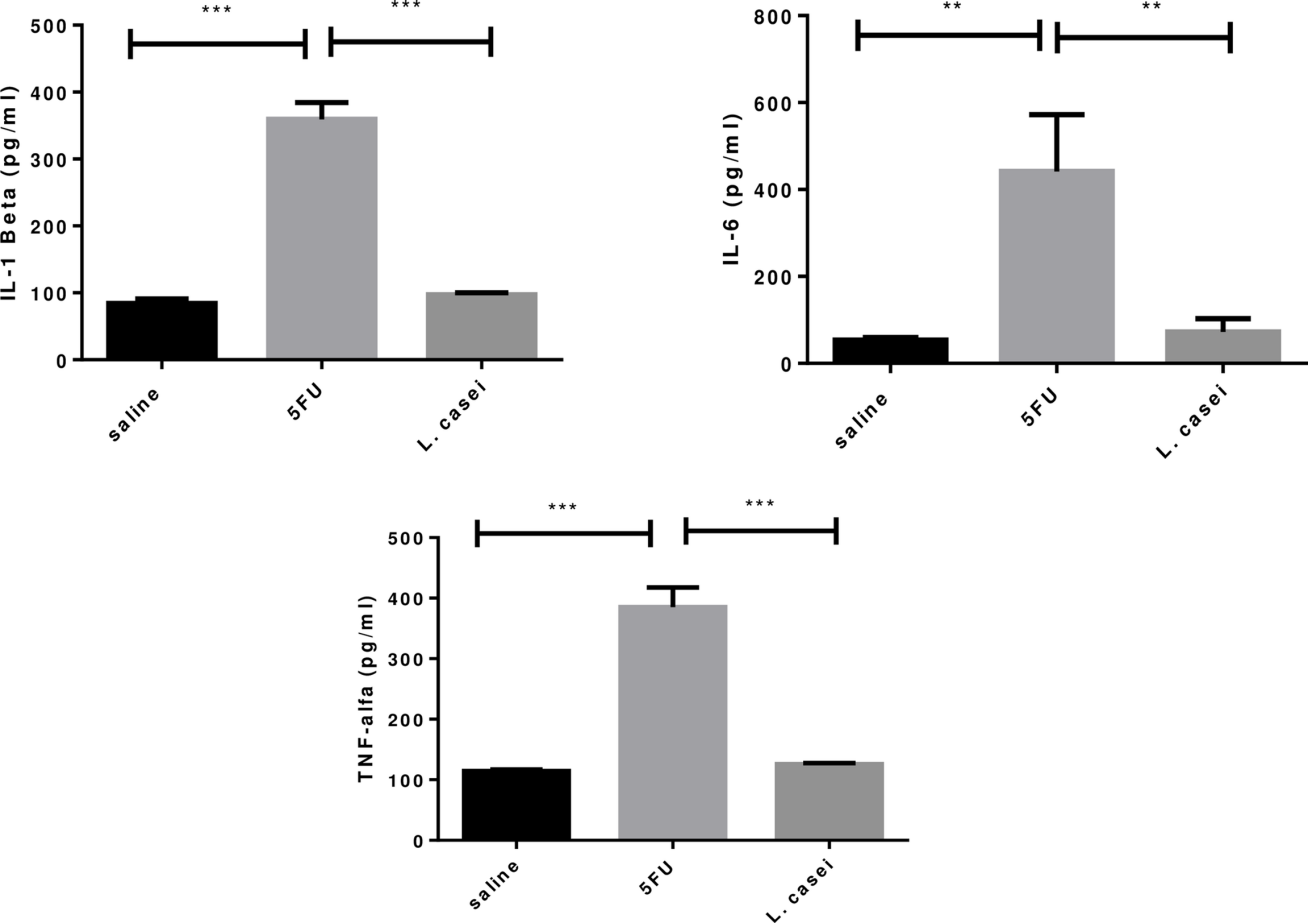
IL-1 beta, IL-6, TNF-α levels. Groups: Saline, 5FU (5-fluorouracil) and L. Casei (*Lacticaseibacillus case*). **p < 0.01, ***p < 0.001.

IL-1 beta, IL-6 and TNF-α levels in the jejunal tissues of animals submitted to 5-FU-induced intestinal mucositis were significantly increased (p<0.05) when compared to the saline group. Pretreatment with *L. casei* significantly reduced (p<0.05) the 5-FU-induced increase in these inflammatory cytokines, restoring these parameters to the level of their respective controls in the saline group, as shown in **Figure 6**.

### *L. casei* protects against 5-FU-induced upregulation of NFKB-P65 and TLR-4 gene expressions and 5-FU-induced downregulation of MUC-2, occludin and zonula occludin gene expressions

**Figure 7:**
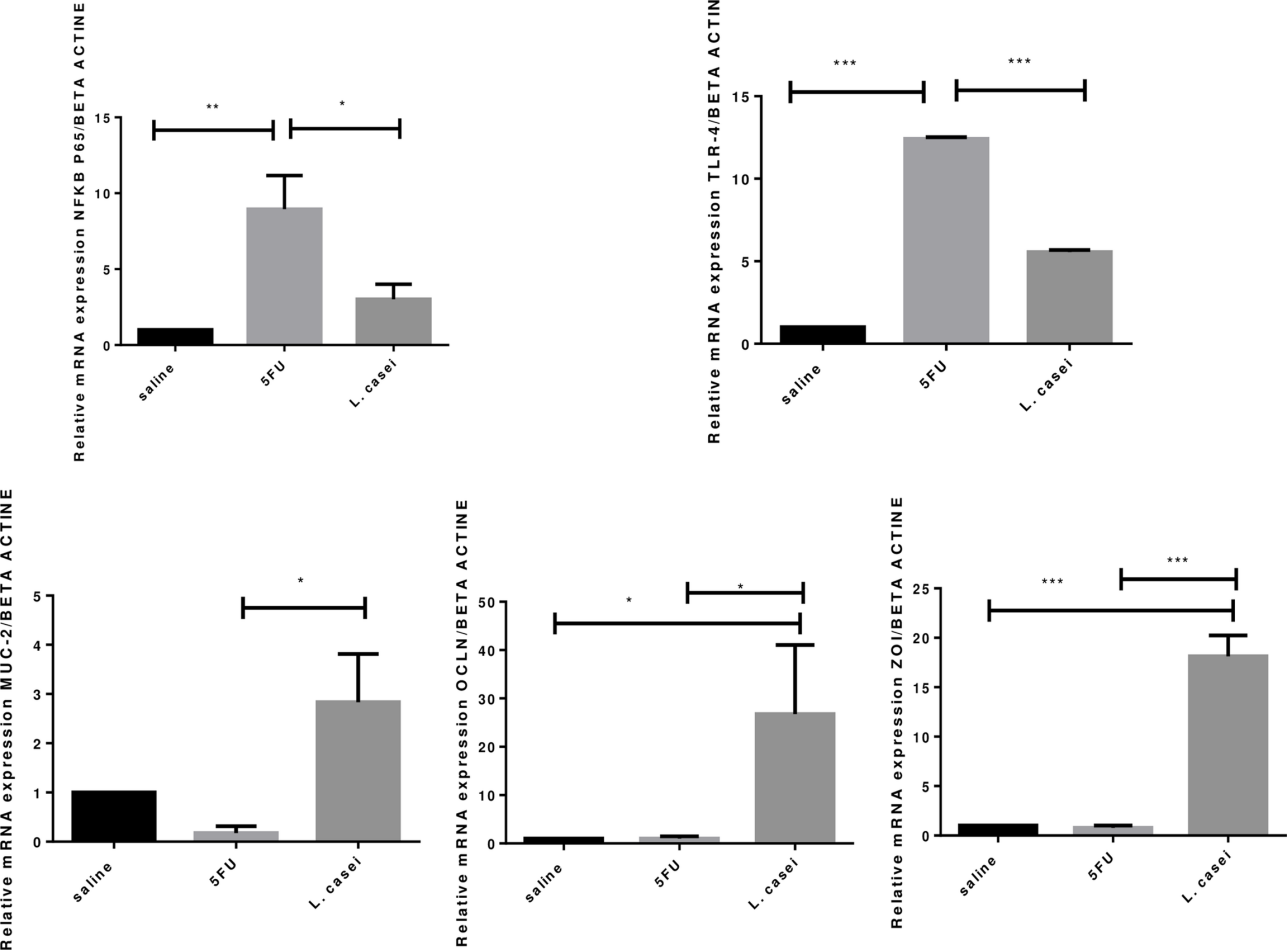
Quantification of gene expression. Beta actin, NFKB-P65, TLR-4, MUC-2 (mucine), OCLN (ocludine), ZO-1 (zonula occluden). Groups: Saline, 5FU (5-fluorouracil) and *L. Casei* (*Lacticaseibacillus casei*). *p < 0.05, **p < 0.01, ***p < 0.001.

RT-PCR analysis showed significant increase in NFKB-P65 and TLR-4 gene expressions in the 5-FU group when compared to the saline group. Pretreatment with *L. casei* was able to protect against these 5-FU effects, as illustrated in **Figure 7**, which shows that *L. casei* significant down-regulated TLR-4 (p<0.05) and NFKB-P65 gene expressions. **Figure 7** also shows that *L. casei* up-regulated MUC-2 (p<0.05), occludin (p <0.05) and ZO-1 (p<0.05), compared to the 5-FU group.

### *L. casei* protects against the effect of 5-FU, increasing fecal lactic acid bacteria

Lactic Acid Bacteria (LAB) were isolated from feces of saline, 5-FU and *L.casei* groups, 7 and 18 days after the first *L.casei* administration. The values, expressed as mean ± SD, are significantly increased (p<0.05) in the *L. casei* group, on days 7 (8.3 ± 0.3) and 18 (8.3 ± 0.3), when compared to both saline (6.4 ± 0.1 and 6.4 ± 0.2, respectively) and 5-FU (6.9 ± 0.1 and 6.5 ± 0.1, respectively) groups. No significant differences were observed between saline and 5-FU groups at any of the evaluated times.

## Discussion

This study provides evidence that *L. casei* protects mice against intestinal damage induced by 5-fluorouracil (5-FU). Other *Lactobacillus* strains were found to be protective (21, 22), or partially protective (23) in experimental 5-FU-induced intestinal injury. In contrast, the ineffectiveness of probiotics in preventing the deleterious effects of 5-FU in mice has also been reported. Whitford et al. (24) demonstrated that *Streptococcus thermophilus* was unable to ameliorate 5-FU induced mucositis (24). Interestingly, their results suggest that it still may have a therapeutic potential effect on oncologic patients, as it was able to decrease mitotic activity and reduce crypt fission, both anti-cancer effects. Mauger et al. (25), using the noninvasive sucrose breath test, reported that *Lactobacillus fermentumBR11*, *Lactobacillus rhamnosusGG*, or *Bifidobacterium lactisBB12* were not able to reduce the severity of small intestinal mucositis at the tested dose (25). We believe that these conflicting results may be attributed to differences between lactobacilli strains, manufacturing conditions and to the influence of methodological choices adopted across studies.

In the current study, pretreatment with *L. casei* was unable to protect against 5-FU-induced weight loss, probably because it is a consequence of anorexia and dehydration commonly associated with 5-FU treatment (26). On the other hand, protective effect of *L. casei* on leukopenia was found, a common side effect of 5-FU. This systemic effect of *L. casei* may have contributed to alleviate 5-FU-induced intestinal inflammation, since leukocytes represent defense cells and can protect animals against bacterial and fungal infections, commonly associated with mucositis (27). In accordance to our results, previous study (28) has shown that oral *L. casei* administration for 5 or 15 days reversed cyclophosphamide-induced immunosuppression in mice by increasing blood red and white blood cell levels and splenocyte and bone marrow cell counts. The beneficial effects of *Lactobacillus delbrueckii* on 5-FU-induced leukopenia in mice has also been demonstrated (21).

The experimental model used in the present study does not allow us assessing whether *L. casei* interferes with 5-FU efficacy. A recent study, however, demonstrated that *Lactobacillus*-derived metabolites enhance the antitumor activity of 5-FU and inhibit metastatic behavior in 5-FU-resistant colorectal cancer cells by regulating claudin-1 expression (29). Accordingly, the analysis of cell adhesion-related gene expression shows significant up-regulation of occludin and zonula occludens-1 (ZO-1), important tight junction proteins, in the intestine of the *L. casei* group. Our results are in agreement with previous studies, showing the role of lactobacilli on the intestinal epithelial barrier regulation (30, 31).

Mechanisms involved in the pathogenesis of mucositis are very complex. Damage to cells due to chemotherapy results in multiple inflammatory events, causing activation of toll like receptors (TLRs), especially TLR4 (32). This elicits upregulation and release of IL-1β, IL-6 and TNF-α *via* the transcription of nuclear factor-κB (NF-κB) (1). In addition to proinflammatory cytokines, the production of large amounts of nitric oxide by inducible nitric oxide enzyme (iNOS) has shown to play a major role in 5-FU- and radiation-induced mucositis, suggesting the important role of reactive oxygen species in the pathogenesis of oral mucositis (33).

In the present study, *L. casei* was able to protect against all 5-FU deleterious effects. Notably, pretreatment with *L. casei* ameliorated the histopathological alterations in the small intestine and colon, associated with reduced IL-1, IL-6 and TNF-α levels, reduced MDA accumulation, an oxidative stress marker, and decreased iNOS and TNF-α protein expressions in jejunal tissues. According to our results, Tang et al. found that a mixture of four probiotic strains ameliorated 5-FU-induced intestinal mucositis and dysbiosis in mice by reducing proinflammatory cytokines, neutrophil infiltration and TLR2 and TLR4 mRNA expression (34). In addition, it has been reported that probiotic bacteria are capable of producing antioxidant compounds that contribute to mitigating the effects of oxidative stress associated with inflammatory bowel disease (21, 35).

Greater lactic acid bacteria population in the feces of the *L. casei* group was observed, compared to both saline and 5-FU control groups. Lactic acid bacteria is a general term for a class of non-spore forming, gram-positive bacteria, whose main product of fermented sugar is lactic acid, a health-regulating probiotic metabolite (36). A clinical trial has demonstrated that humans consuming *Lactobacillus Rhamnousus* had increased concentration of the probiotic in feces (37). Another study (38) also observed increase in viable lactobacilli in feces during *Lactobacillus acidophilus* administration, persisting for at least 7 days after the end of supplementation.

The beneficial effects of *L. casei* observed in the present study may be associated with normalization of intestine microbiota, previously disrupted by 5-FU (34). It has been well established that intestinal microbiota acts in symbiosis to modulate different functions, such as the stimulation-regulation of epithelial innate immunity and competitive adherence to the mucosa and epithelium, acting as a true barrier to aggressive agents (6).

Microbiota analysis was not performed, which is a limitation for this study, but a significant decrease in the invertase activity was found in the intestine of mice submitted to 5-FU-induced intestinal mucositis. This finding may be associated with 5-FU-induced dysbiosis, since invertase is produced by microorganisms (39). *L. casei* was not able to preserve the invertase activity. However, significant and greater increase in the mucin gene expression was found in the intestine of *L. casei* group compared to both saline and 5-FU groups. Strong link between bacteria (intestinal flora) and mucin secretion has been reported, both of which being shown to be affected in chemotherapy-induced mucositis (40).

Mucins, the main components of the intestinal epithelium surface, play an important role in the integrity of the gut microbiota by providing attachment sites for intestinal flora and pathogenic bacteria, as well as simultaneously protecting the mucosa from bacterial overgrowth and/or penetration (40).

There are limitations in this study that should be discussed. Further studies are needed to investigate the gut microbiota variation after 5-FU administration, as well as the impact of *L. casei*. In addition, although probiotic therapy seems to be safe and risk-free, more animal studies should be developed to assess the safety and efficacy of *L. casei*, prior to its introduction as a therapy in clinical practice.

The results of this study show that oral *Lacticaseibacillus casei* administration has beneficial effects on 5-FU-induced mucositis, acting on the modulation of genes related to the barrier function, controlling the levels of pro-inflammatory cytokines and considerably attenuating the damage to the mucosa caused by 5-FU. Thus, new perspectives are opened for the use of *Lacticaseibacillus casei* as an alternative strategy for the prevention or management of chemotherapy-induced mucositis in the future.

## Data availability statement

The original contributions presented in the study are included in the article/supplementary material. Further inquiries can be directed to the corresponding authors.

## Ethics statement

The animal study was reviewed and approved by Ethics Committee on the use of Animals (CEUA) of Federal University of Rio Grande do Norte.

## Author contributions

SB, MO, GG, FS, AA conceived and planned the experiments. SB, MO, SR, CM, ML, GG, RA, FS, AM, DF, RA, CR, GB, RL, AA carried out the experiments. SB, MO, SR, CM, ML, GG, RA, FS, AM, DF, RA, CR, GB, RL, AA contributed to sample preparation. SB, MO, SR, CM, ML, GG, RA, FS, AM, DF, RA, CR, GB, RL, AA contributed to the interpretation of the results. SB, MO, CM, GG, FS, CR, GB, RL, AA took the lead in writing the manuscript. All authors provided critical feedback and helped shape the research, analysis and manuscript. All authors contributed to the article and approved the submitted version.

## Funding

This study was supported by the Conselho Nacional de Desenvolvimento Científico e Tecnológico (CNPQ) Number: 304382/2020-5, PROPLAN Number 184/2020. This study was financed in part by the Coordenação de Aperfeicoamento de Pessoal de Nível superior -Brasil (CAPES)-Finance Code 001.

## Conflict of interest

The authors declare that the research was conducted in the absence of any commercial or financial relationships that could be construed as a potential conflict of interest.

## Publisher’s note

All claims expressed in this article are solely those of the authors and do not necessarily represent those of their affiliated organizations, or those of the publisher, the editors and the reviewers. Any product that may be evaluated in this article, or claim that may be made by its manufacturer, is not guaranteed or endorsed by the publisher.
